# Exploring the relationship between sepsis and Golgi apparatus dysfunction: bioinformatics insights and diagnostic marker discovery

**DOI:** 10.3389/fgene.2025.1483493

**Published:** 2025-02-06

**Authors:** Wanli Ma, Xinyi Liu, Ran Yu, Jiannan Song, Lina Hou, Ying Guo, Hongwei Wu, Dandan Feng, Qi Zhou, Haibo Li

**Affiliations:** ^1^ Department of Anesthesiology, Municipal Hospital of Chifeng, Chifeng, Inner Mongolia, China; ^2^ Department of Anesthesiology, Chifeng Clinical College of Inner Mongolia Medical University, Chifeng, Inner Mongolia, China

**Keywords:** sepsis, Golgi apparatus, immune infiltration, signature, gene co-expression network

## Abstract

**Background:**

Sepsis, a critical infectious disease, is intricately linked to the dysfunction of the intracellular Golgi apparatus. This study aims to explore the relationship between sepsis and the Golgi apparatus using bioinformatics, offering fresh insights into its diagnosis and treatment.

**Methods:**

To explore the role of Golgi-related genes in sepsis, we analyzed mRNA expression profiles from the NCBI GEO database. We identified differentially expressed genes (DEGs). These DEGs, Golgi-associated genes obtained from the MSigDB database, and key modules identified through WGCNA were intersected to determine Golgi-associated differentially expressed genes (GARGs) linked to sepsis. Subsequently, functional enrichment analyses, including GO, KEGG, and GSEA, were performed to explore the biological significance of the GARGs.A PPI network was constructed to identify core genes, and immune infiltration analysis was performed using the ssGSEA algorithm. To further evaluate immune microenvironmental features, unsupervised clustering was applied to identify immunological subgroups. A diagnostic model was developed using logistic regression, and its performance was validated using ROC curve analysis. Finally, key diagnostic biomarkers were identified and validated across multiple datasets to confirm their diagnostic accuracy.

**Results:**

By intersecting DEGs, WGCNA modules, and Golgi-related gene sets, 53 overlapping GARGs were identified. Functional enrichment analysis indicated that these GARGs were predominantly involved in protein glycosylation and Golgi membrane-related processes. PPI analysis further identified eight hub genes: B3GNT5, FUT11, MFNG, ST3GAL5, MAN1C1, ST6GAL1, C1GALT1C1, and GALNT14. Immune infiltration analysis revealed significant differences in immune cell populations, mainly activated dendritic cells, and effector memory CD8^+^ T cells, between sepsis patients and healthy controls. A diagnostic model constructed using five pivotal genes (B3GNT5, FUT11, MAN1C1, ST6GAL1, and C1GALT1C1) exhibited predictive accuracy, with AUC values exceeding 0.96 for all genes. Validation with an independent dataset confirmed the differential expression patterns of B3GNT5, C1GALT1C1, and GALNT14, reinforcing their potential as robust diagnostic biomarkers for sepsis.

**Conclusion:**

This study elucidates the link between sepsis and the Golgi apparatus, introduces novel biomarkers for sepsis diagnosis, and offers valuable insights for future research on its pathogenesis and treatment strategies.

## 1 Introduction

Sepsis, characterized as a systemic inflammatory response syndrome triggered by infection, can precipitate organ dysfunction and shock (Singer et al., 2016). It stands as the predominant cause of mortality within hospital settings and represents a significant public health challenge worldwide (Li et al., 2023). Sepsis, a grave infectious condition, exhibits a notably intricate pathogenesis that involves various biological processes and the malfunctioning of organelles. Primarily, bacterial infections, particularly those caused by Gram-negative bacteria, are identified as the chief instigators of sepsis (Martin, 2014). Bacterial endotoxins, such as lipopolysaccharide, instigate the activation of the host’s immune response, culminating in a systemic inflammatory reaction. This inflammatory cascade is propelled by a sequence of signal transduction pathways and the release of inflammatory mediators, including tumor necrosis factor-alpha (TNF-α) and interleukin-1 beta (IL-1β). Historical research has predominantly concentrated on identifying the source of infection, the pathogens involved, and the patient’s immune response (Huang et al., 2019). Nonetheless, recent investigations have illuminated the critical role of the Golgi apparatus in the pathogenesis and progression of sepsis.

The Golgi apparatus, a crucial organelle within eukaryotic cells, resides in the cytoplasm and consists of membranous vesicles (Hicks and Machamer, 2005). It forms a continuous membrane system with the endoplasmic reticulum (Casey et al., 2018). This organelle plays a vital role in protein synthesis, modification, and distribution, and in the metabolism of lipids and sugars. Additionally, it participates in various biological processes, including cell signaling, division, and apoptosis. A notable pathological characteristic of the Golgi apparatus is its structural disintegration (Choi et al., 2022). Under conditions of intracellular homeostasis, its distinctive stacked structure remains intact, and its dynamic functions are meticulously regulated (Jiang et al., 2011). Conversely, structural modifications to the Golgi can precipitate or aggravate disease conditions. Dysfunctional Golgi activity has been associated with the development and progression of numerous diseases, such as cancers, neurodegenerative disorders, and inflammatory conditions. This association extends to physiological processes like intracellular calcium ion balance, oxidative stress, and cell death. The impact of sepsis on the Golgi apparatus may be mediated through various mechanisms, including the production of reactive oxygen species (ROS), influx of calcium, activation of proteasome pathways, and induction of apoptosis (Jiang et al., 2011). Specifically, Golgi damage can undermine its critical roles in cell signaling, protein synthesis, and secretion (Liu et al., 2021). Recent investigations using animal models of sepsis have demonstrated that Golgi dysfunction can heighten apoptosis and inflammatory responses (Ma et al., 2008). In models of acute lung injury, the Golgi stress response has been shown to mitigate lung damage via the HIF-1α/HO-1 signaling pathway (Li et al., 2021). HO-1 mediates ferritin deposition in the macrophage subtype Kupffer cells, driving immune-inflammatory responses (Li et al., 2024). Therefore, the response to Golgi stress could be pivotal in determining cell fate by modulating Golgi apparatus changes. In the context of infection, disruptions in the Golgi structure may facilitate evasion of host immune detection (Derré et al., 2020).

Understanding the molecular interactions between sepsis and the Golgi apparatus necessitates further research. Unraveling this relationship could unveil novel therapeutic targets for combating sepsis.

In our study, we sourced genetic data from both septic and normal blood samples using the GEO database to identify differentially expressed genes (DEGs) between the two conditions. We applied Weighted Gene Correlation Network Analysis (WGCNA) to pinpoint the gene modules most pertinent to sepsis, leading to the identification of DEGs linked to the Golgi apparatus. Additionally, we analyzed immune cell infiltration within both septic and control groups employing the ssGSEA algorithm. Our research culminated in the screening for potential diagnostic markers of sepsis. By elucidating the connection between the Golgi apparatus and sepsis, this study aims to enhance our comprehension of sepsis’ pathological mechanisms and inspire novel diagnostic and therapeutic strategies.

## 2 Methods

### 2.1 Acquisition of microarray data and identification of differentially expressed genes

Sepsis-associated transcriptome datasets GSE95233 and GSE57065 were retrieved from the NCBI Gene Expression Omnibus (GEO) database (Barrett et al., 2013). The GSE57065 dataset, generated on the Affymetrix Human Genome U133 Plus 2.0 Array (GPL570 platform), includes data from 25 normal individuals and 82 sepsis patients (Tabone et al., 2019). Similarly, GSE95233 was also produced on the GPL570 platform comprised 22 normal and 51 sepsis patient samples (Venet and Monneret, 2017). These datasets were combined using the R package “sva,” with batch effects mitigated through normalization techniques and the ComBat method within the same package (Leek et al., 2012). The consolidated dataset categorized samples into normal (n = 47) and sepsis (n = 133) groups. Differential gene expression analysis was conducted using the “limma” package in R, applying thresholds of logFC >1 or < −1 and an adjusted P-value <0.05 (Ritchie et al., 2015). Visualization of differentially expressed genes (DEGs) was achieved using “ggplot2” for the volcano and box plots and “ComplexHeatmap” for heatmaps.

The gene set “GOCC_GOLGI_APP,” which pertains to Golgi-related genes, was identified and downloaded from the Gene Set Enrichment Analysis (GSEA) MSigDB database (Liberzon et al., 2015).

### 2.2 Weighted gene correlation network analysis (WGCNA)

WGCNA is a systematic biological method widely utilized to delineate genetic association patterns among various samples, elucidate gene network-phenotype relationships, and identify core network genes. This approach facilitates the identification of potential markers through genomic correlations and genome-phenotype associations. It involves constructing hierarchical clustering trees based on correlation coefficients, where branches represent different gene modules, each color-coded for distinction. Using the “WGCNA” package in R software (Langfelder and Horvath, 2008), we constructed gene co-expression networks for groups with and without sepsis. We selected the top 5,000 genes with the highest expression variability (coefficient of variation >0.5) for analysis and applied a soft threshold power of 6 (scale-free R^2^ > 0.85) to compute a weighted adjacency matrix, subsequently transformed into a topological overlap matrix (TOM). Modules were identified using the dynamic tree cut method (minimum module size = 30) and merged if the module eigengene similarity exceeded 0.25. Modules significantly associated with sepsis (*P* < 0.05) were selected for further analysis. Subsequently, we assessed the relevance of different modules to sepsis pathogenesis, identifying the most pertinent modules as central genes through WGCNA. The intersection of differentially expressed genes, Golgi-associated genes, and WGCNA-identified central genes were designated as Golgi-associated differential genes (GARGs), offering new insights into the genetic underpinnings of sepsis.

### 2.3 Functional annotations for GARGs

To elucidate the roles of differentially expressed GARGs, we conducted Gene Ontology (GO) (Gene Ontology, 2015) and Kyoto Encyclopedia of Genes and Genomes (KEGG) (Wrzodek et al., 2011) pathway enrichment analyses using the “clusterProfiler” R package (Yu et al., 2012). The significance threshold was set at a P-value <0.05, employing the Benjamini-Hochberg correction for multiple comparisons. GO analysis facilitated the large-scale functional categorization of genes into molecular functions (MF), biological processes (BP), and cellular components (CC). KEGG provided insights into biological pathways, pharmaceutical drugs, and genomic information. Visualization of the results was achieved through Chord and Circle plots, utilizing “ggplot2” and “GOplot” R packages, respectively. Additionally, gene set enrichment analysis (GSEA) was performed on the “c5.go.v7.4.symbols” gene set from the MSigDB database with “clusterProfiler” where results with a P-value <0.05 were deemed statistically significant and visualized using “ggplot2.”

### 2.4 Protein-protein interaction (PPI) analysis and hub gene identification

Initial PPI analysis of Golgi-related genes was executed using the STRING database, setting a composite score threshold of ≥0.4 (Doncheva et al., 2019). The resulting data, in TSV format, was imported into Cytoscape 3.10.0 for network graph visualization (Szklarczyk et al., 2021). Subsequent identification of significant hub genes (Hub-GARGs) within the network was facilitated by the MCODE and CytoHubba plugins in Cytoscape, enhancing the analysis and filtering process.

### 2.5 Prediction of transcription factor (TF) networks and miRNA networks

The NetworkAnalyst online platform was employed to construct a gene regulatory network, focusing on interactions between hub genes and transcription factors (Castro-Mondragon et al., 2022). This involved utilizing TF and gene target information from the JASPAR (Xia et al., 2015) database and visualizing the Hub-GARGs-TF network in Cytoscape 3.10.0. Hub-GARGs were further analyzed against four databases, including the miRTarBase database, using R software to validate and predict relationships with relevant miRNAs (Huang et al., 2022). The final network visualization was conducted in Cytoscape.

### 2.6 Immune infiltration analysis

We analyzed the infiltration of 28 immune cell types in normal and septic samples using the “ssGSEA” method within the R package, identifying significant differences at *P* < 0.05 (Chen et al., 2018). The ssGSEA analysis was performed using the “GSVA” package with method = “ssgsea” and input data normalized using log2 transformation and Z-score scaling. Additional immune infiltration analyses, including Xcell, QuanTiseq, and MCP-counter, were conducted using the “IOBR” package for a more comprehensive assessment (Zeng et al., 2021). Box plots were utilized to delineate the disparities in immune cell distributions between normal individuals and sepsis patients. Concurrently, we undertook a correlation analysis of these 28 immune cell types using R software. To delve deeper into the relationships among immune cells and their association with Hub-GARGs, Spearman correlation analysis was conducted using the “Corrplot” function in R. Significance thresholds were set at *P* < 0.05.

### 2.7 Recognition of distinct immune microenvironment subtypes by unsupervised clustering

Using the expression profiles of sepsis and ssGSEA scores, the number of unsupervised clusters in a sample of sepsis was quantitatively assessed through consensus clustering, implemented with the ConsensusClusterPlus software package (50 iterations and an 80% resampling rate). Key metrics, including the cumulative distribution function (CDF) curve performance, the consensus matrix, the relative change in the area under the CDF curve, and the consensus clustering score (>0.9), were evaluated. The number of clusters (k) was tested between 2 and 9, with the optimal k value determined when the CDF index approached its maximum. Heatmaps and box plots were utilized to visualize immune cell expression across immune subtypes, while violin plots illustrated the differential expression of Hub-GARGs among the immune subtypes.

### 2.8 Construction of diagnostic models

Logistic regression was employed to isolate the most potent prognostic indicators, which is designed to prevent model overfitting by constraining the absolute values of the regression coefficients. The “glmnet” package was used, and the Least Absolute Shrinkage and Selection Operator (LASSO) technique was applied for preliminary gene screening related to sepsis. Subsequently, a diagnostic model was constructed through logistic regression, calculating the odds ratio (OR) and P-value for each variable to derive a risk score for each sample. Diagnostic marker genes selected had *P* < 0.05 and OR values indicating significance. A nomogram model was then developed to forecast the risk of sepsis utilizing the “rms” software package (Park, 2018), and the diagnostic efficacy of the candidate biomarkers was assessed by generating a Receiver Operating Characteristic (ROC) curve with the ROC software package. The accuracy of the model was quantified using the area under the ROC curve (AUC).

### 2.9 Validation of key Golgi genes

The GSE26378 dataset, sourced from the GEO database and generated on the Affymetrix Human Genome U133 Plus 2.0 Array (GPL570 platform), comprises data from 21 normal and 81 sepsis patient samples (Wynn et al., 2011). The expression patterns and distributions of Golgi-related genes were validated and visualized using heatmaps and box plots with the “ggplot2” R package.

## 3 Results

### 3.1 Identification and analysis of differential genes in sepsis

**FIGURE 1:**
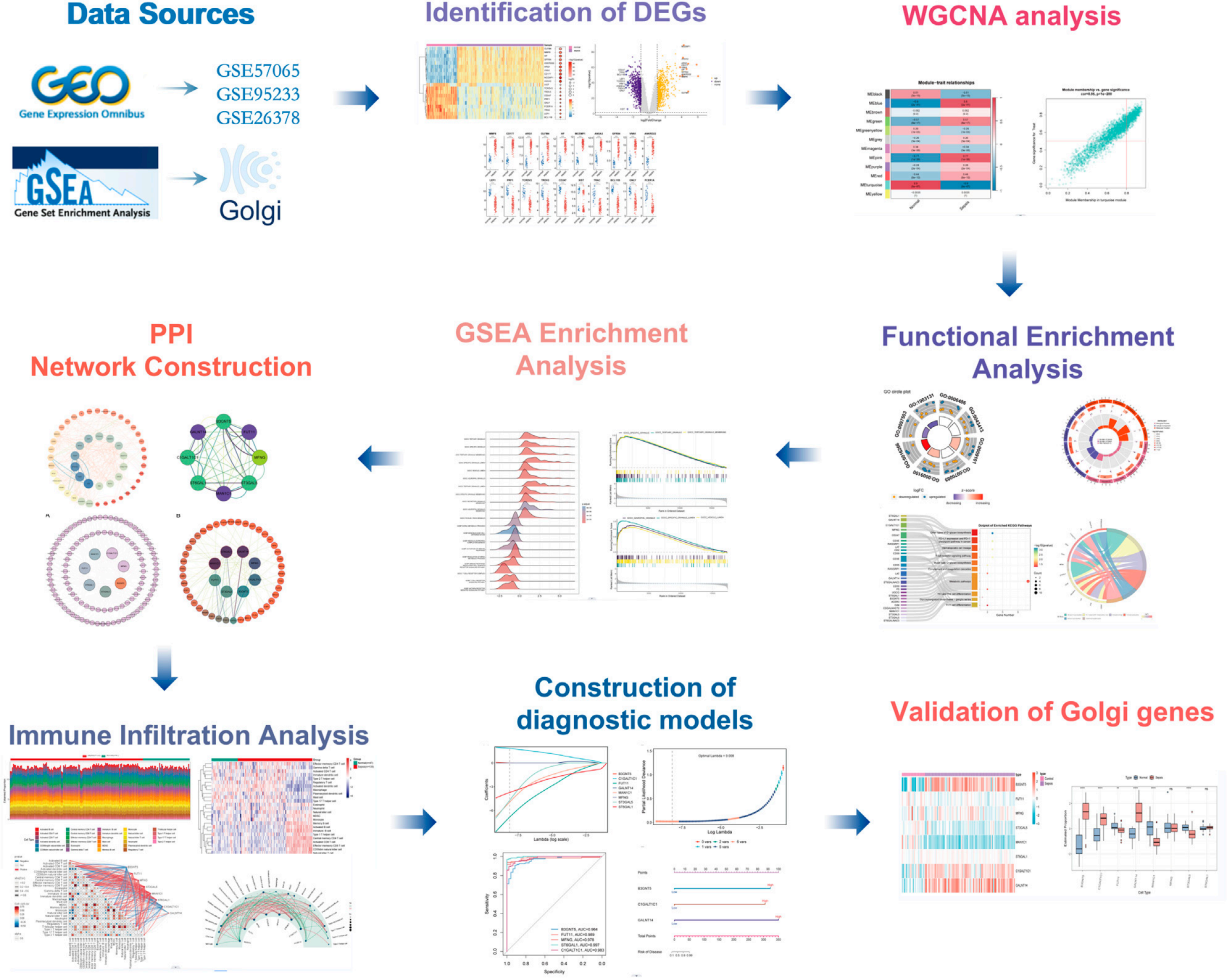
Flowchart of the multistep screening strategy on bioinformatics data.

**FIGURE 2:**
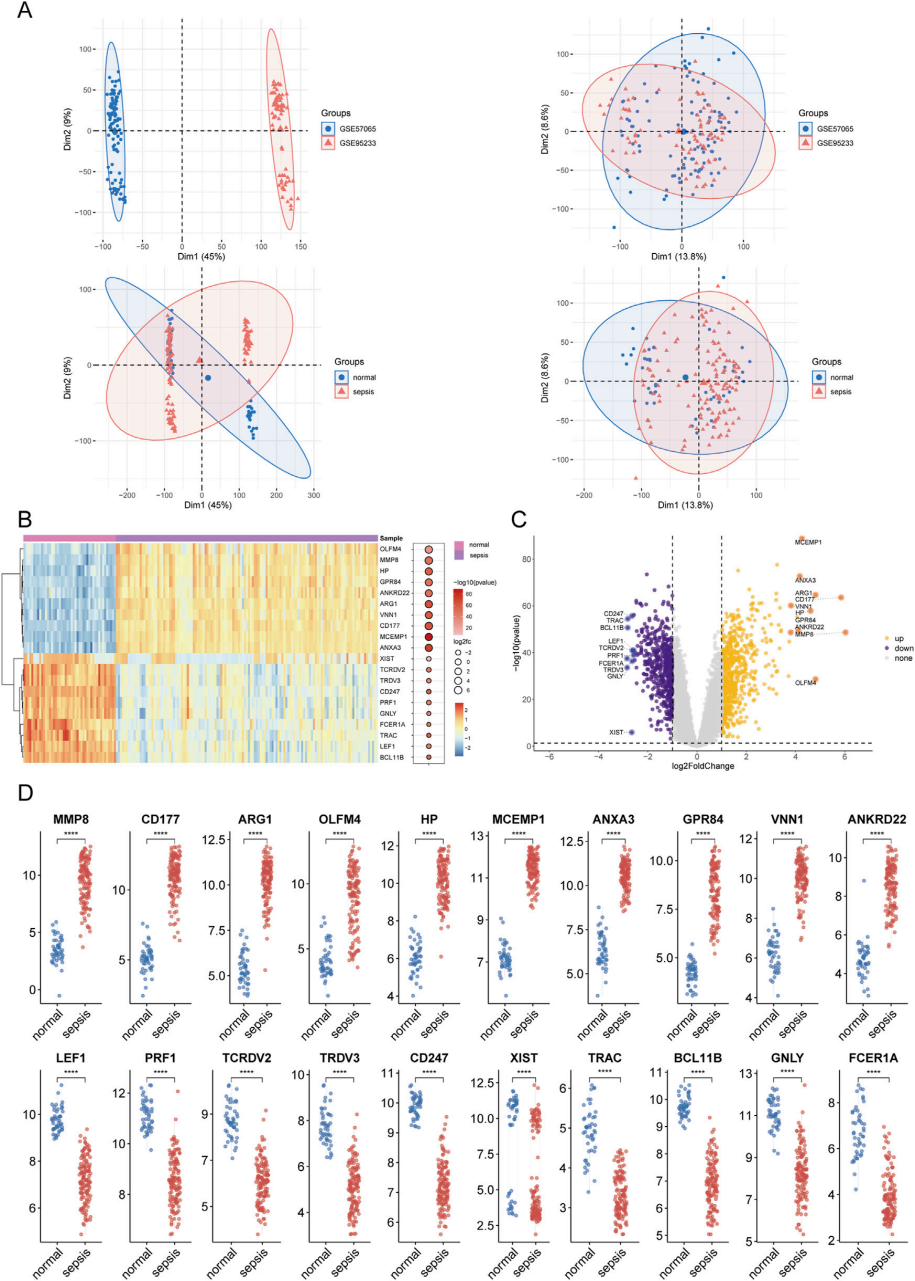
Results of differential expression analysis in sepsis. **(A)** Principal component analysis (PCA) plot illustrating the dataset after batch effect correction. **(B, C)** Clustered heatmaps and volcano plots displaying differentially expressed genes (DEGs) between sepsis and control groups. Upregulated genes are shown in red, and downregulated genes are shown in blue, with significant thresholds set at adjusted *P* < 0.05 and |log2FC|>1. **(D)** Box plot summarizing the expression levels of selected DEGs. Asterisks denote statistical significance: *P < 0.05, **P < 0.01, ***P < 0.001, ****P < 0.0001.

The methodological framework of this study is depicted in Figure 1. Datasets GSE9960 and GSE57065 were retrieved from the GEO database and combined, with batch effects subsequently mitigated. Analysis of data distribution and pre- and post-batch effect mitigation was conducted using PCA plots. This analysis aimed to assess potential disparities in the expression profiles of the two datasets. Initially, distinct differences between the datasets were evident. However, following the adjustment for batch effects, these disparities were no longer observable (Figure 2A). Differential expression analysis, facilitated by the “limma” package in R, identified 1,222 DEGs between sepsis and control groups. This comprised 661 upregulated and 561 downregulated genes (Figures 2B–D). Additionally, a gene set encompassing 1,659 GARGs was acquired from the GSEA database (GOCC_GOLGI_DATA), enriching the study’s resources.

### 3.2 Construction of WGCNA network and identification of GARGs

**FIGURE 3:**
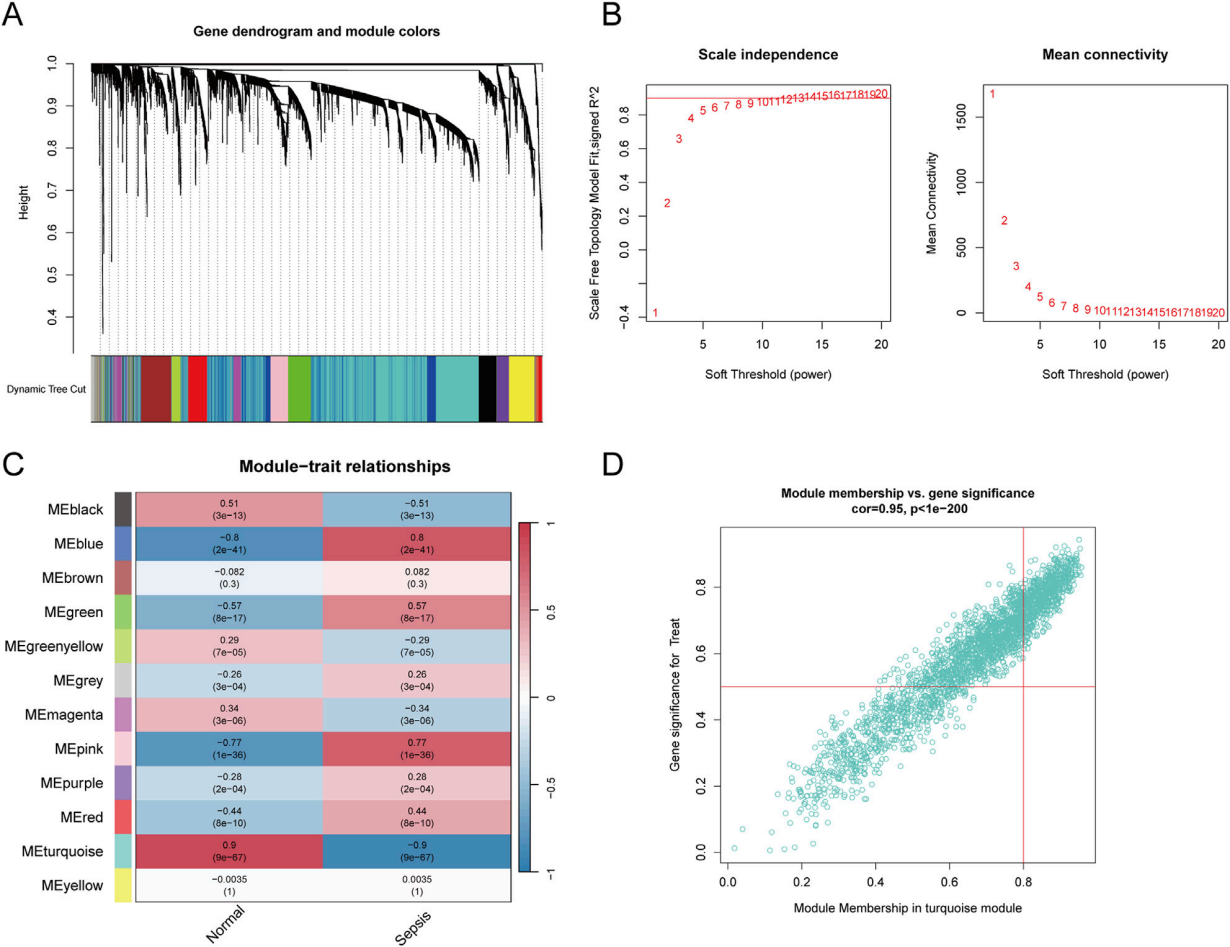
Weighted gene co-expression network analysis. **(A)** Gene clustering dendrogram based on the topological overlap, with dynamic tree cutting to identify modules represented by different colours. **(B)** Soft threshold selection and corresponding scale-free topology fit index and mean connectivity, with the optimal soft threshold chosen to maintain scale-free topology. **(C)** Heatmap showing module-trait correlations, illustrating the association between identified gene modules and sepsis. The colour intensity indicates the strength and direction of the correlation. **(D)** Scatter plot of module membership versus gene significance in the turquoise module, showing a strong correlation with sepsis.

**FIGURE 4:**
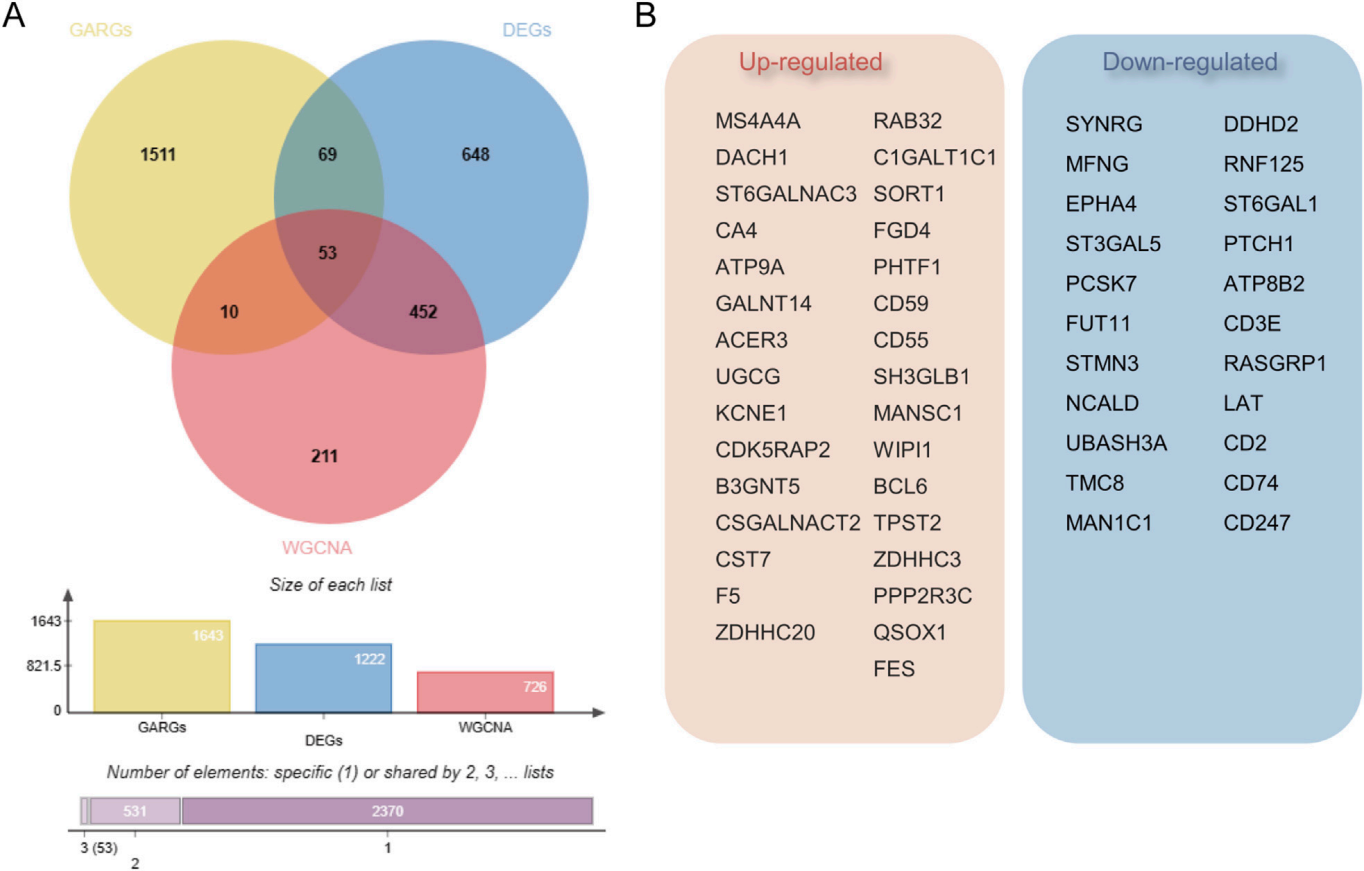
Identification of Golgi-associated related genes (GARGs). **(A)** Venn diagram showing the overlap of GARGs, DEGs, and WGCNA-identified genes. A total of 53 overlapping genes were identified as Golgi-related DEGs, with additional intersections highlighted. The bar plot indicates the size of each gene set and their overlaps. **(B)** The 53 overlapping genes include 31 upregulated and 22 downregulated genes, reflecting their differential expression patterns in Golgi-related pathways in sepsis.

To ascertain if gene modules are associated with sepsis, we executed a Weighted Gene Co-expression Network Analysis (WGCNA) on all candidate genes from the consolidated datasets. This analysis resulted in the identification of 12 distinct modules (Figures 3A–C). Subsequent analysis, focusing on positive correlation coefficients, revealed the Meturquoise module as exhibiting the highest correlation and significant divergence. Hence, genes within the Meturquoise module were earmarked for further examination (Figure 3D). Intersection analysis involving the Meturquoise module, differential genes, and Golgi-related genes yielded 53 Golgi Apparatus-Related Genes (GARGs) (Figure 4).

### 3.3 Functional interpretation of Golgi-related differential genes in sepsis

**FIGURE 5:**
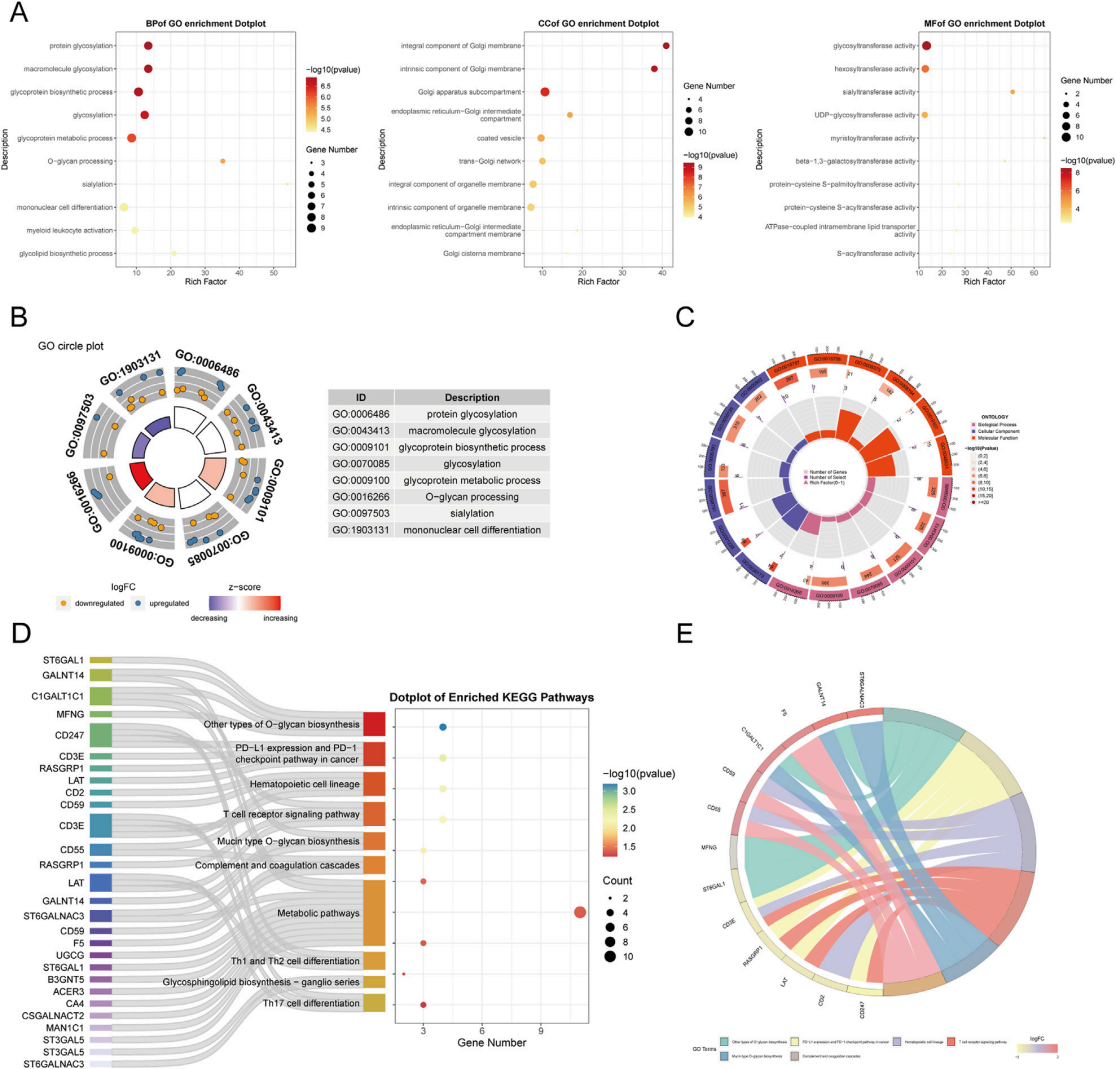
Functional enrichment analysis of GARGs.**(A)** Dot plots of GO enrichment analysis in BP, CC, and MF categories, with dot size representing gene counts and colour indicating significance. **(B)** GO circle plot summarizing top enriched terms. **(C)** The circular heatmap shows Z-scores of the top enriched GO terms, with red and blue indicating upregulated and downregulated trends, respectively. **(D)** Sankey plot showing the mapping of GARGs to enriched KEGG pathways. **(E)** Circos plot illustrating the association between GARGs and their top enriched KEGG pathways, with the width of lines representing the strength of the association.

**FIGURE 6:**
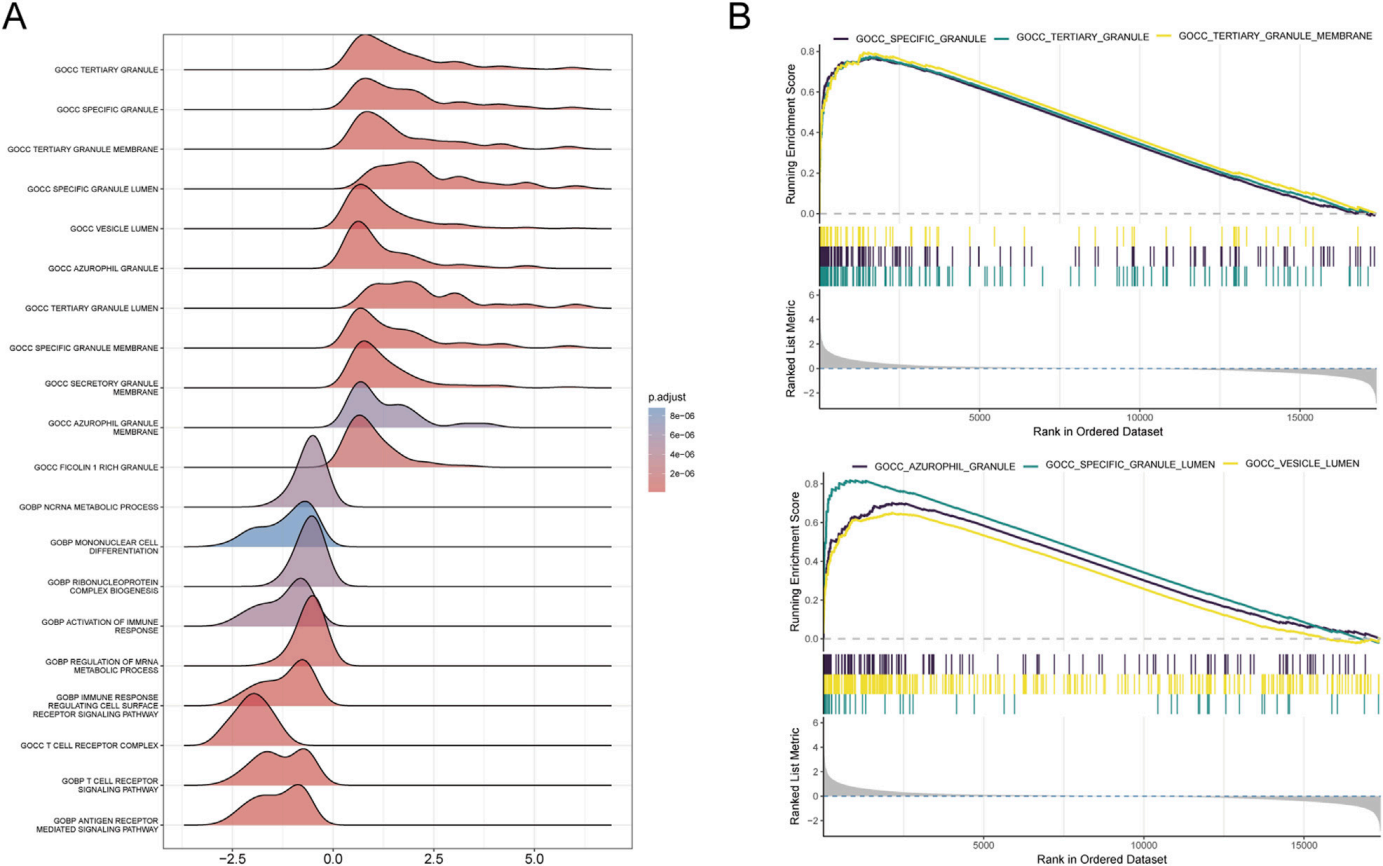
Results of GSEA analysis. **(A)** Ridge plot illustrating the GSEA results for the combined dataset, with each ridge representing an enriched GO term. The colour gradient indicates adjusted P-values, with darker colours representing higher significance. **(B)** Line plots displaying the top six enriched GO terms from the GSEA analysis. Each plot shows the running enrichment score across the ranked dataset, with bars below indicating gene rank positions contributing to the enrichment of the term.

We conducted functional annotation of the intersected genes through GO and KEGG analyses. GO enrichment analysis indicated significant enrichment of genes in biological processes (BPs) related to protein glycosylation, macromolecule glycosylation, and glycoprotein biosynthetic processes. In the cellular component (CC) category, enrichment was observed in the integrative and intrinsic components of the Golgi membrane, as well as Golgi apparatus subcompartments. Molecular function (MF) analysis revealed a predominance of genes involved in glycosyltransferase and hexosyltransferase activities (Figures 5A–C). KEGG enrichment analysis identified significant gene involvement in the biosynthesis of various O-glycans, PD-L1 expression and PD-1 checkpoint pathways in cancer and the hematopoietic cell lineage (Figures 5D, E). Furthermore, Gene Set Enrichment Analysis (GSEA) highlighted the primary enrichment of cellular component genes in specific granule, tertiary granule, and tertiary granule membrane categories (Figures 6A, B).

### 3.4 PPI network construction and identification of Hub-GARGs

**FIGURE 7:**
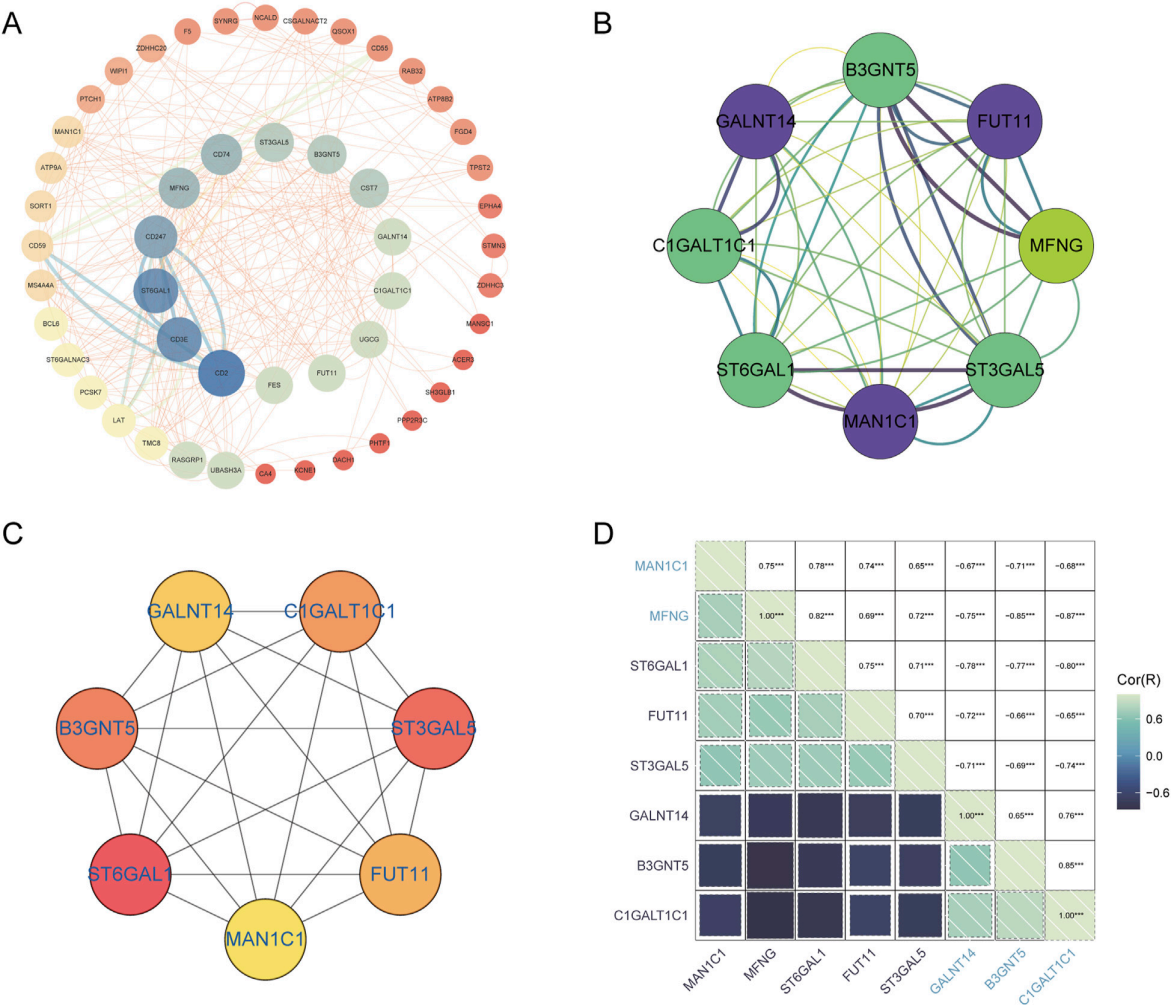
PPI network analysis and identification of hub GARGs. **(A)** A PPI network of GARGs was constructed using the STRING database, showing interactions between Golgi-associated genes. **(B)** A key gene cluster identified by the MCODE plugin in Cytoscape contains 8 hub genes with strong interconnections. **(C)** The top 7 hub genes identified by the CytoHubba algorithm are ranked by their centrality scores. **(D)** Correlation matrix illustrating pairwise correlations between the identified GARGs, with colour intensity representing the strength and direction of the correlation.

Golgi-related differential genes were uploaded to the String database, resulting in the construction of a protein-protein interaction (PPI) network comprising 53 nodes and 155 edges, with an average node degree of 5.85. This was achieved using the String database’s default criteria. The network data were then exported from the String database and visualized using Cytoscape software (Figure 7A). Within Cytoscape, the MCODE plugin was utilized to identify significant modules, leading to the isolation of a network graph featuring eight Golgi-related differential genes (Figure 7B). Subsequently, the CytoHubba plugin, employing the MCC algorithm, pinpointed seven potential hub genes within the PPI network: B3GNT5, FUT11,ST3GAL5, MAN1C1, ST6GAL1, C1GALT1C1, and GALNT14 (Figure 7C). The analysis also included the investigation of interrelations among these hub genes (Figure 7D).

### 3.5 miRNA network and transcription factor network of GARGs

**FIGURE 8:**
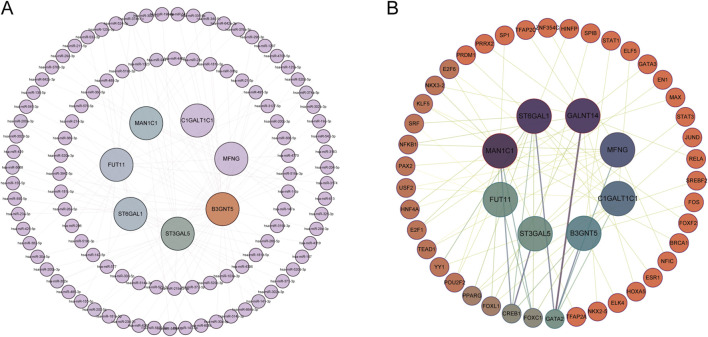
miRNA and TF regulatory networks of GARGs. **(A)** miRNA regulatory network showing interactions between GARGs and their associated miRNAs. The inner nodes represent GARGs, while the outer nodes represent regulatory miRNAs. **(B)** TF regulatory network illustrating the relationships between GARGs and their upstream TFs. The central nodes represent GARGs, and the surrounding nodes indicate interacting TFs. Line colorus indicate the strength and type of regulatory interactions.

Utilizing R, miRNA predictions for Hub-GARGs were conducted across three databases: ENCORI, miRDB, and RNAInter. These analyses identified that hsa-miR-374a-5p is associated with ST3GAL5, FUT11, and B3GNT5. Similarly, hsa-miR-374b-5p showed associations with the same genes: ST3GAL5, FUT11, and B3GNT5 (Figure 8A). Furthermore, the NetworkAnalyst platform facilitated the construction of a gene regulatory network, which comprises 42 transcription factors interacting with the Hub-GARGs network (Figure 8B).

### 3.6 Analysis of immune infiltration in sepsis

**FIGURE 9:**
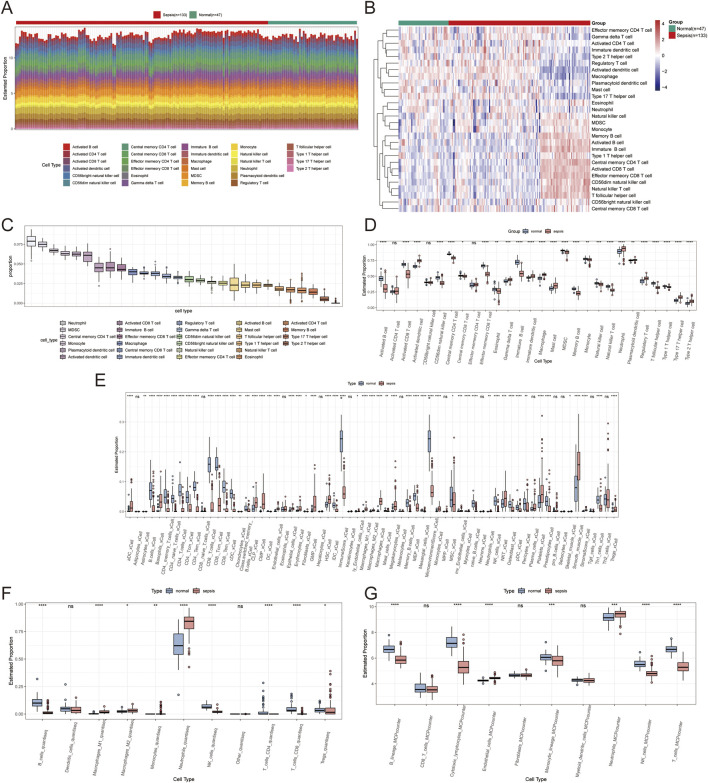
Immune infiltration analysis results. **(A)** A stacked bar plot shows the proportions of immune cell types identified by ssGSEA analysis in normal and sepsis samples. **(B)** Heatmap illustrating immune cell infiltration levels in normal and sepsis samples based on ssGSEA analysis, with colour intensity representing infiltration levels. **(C)** Box plot summarizing the overall distribution of 28 immune cell types identified by ssGSEA analysis. **(D)** Box plots comparing the proportions of immune cells between normal and sepsis groups. **(E)** Box plots of immune cell proportions based on Xcell analysis, highlighting differences between normal and sepsis samples. **(F)** Box plots of immune cell proportions based on QuanTiseq analysis show significant variations across the two groups. **(G)** Box plots of immune cell proportions based on MCP-counter analysis show significant variations across the two groups.

The ssGSEA algorithm was utilized to assess immune cell infiltration across dataset samples to elucidate the variances in immune function associated with sepsis. This analysis revealed significant differences in the infiltration levels of 26 immune cell types between sepsis and control samples (*P* < 0.05), highlighting pronounced disparities in activated dendritic cells, plasmacytoid dendritic cells, and effector memory CD8^+^ T cells (Figures 9A–D). Additionally, Xcell analysis indicated substantial differences in macrophages and CD8^+^ T cells across the two sample groups (Figure 9E). QuanTiseq analysis revealed significant variations in macrophages M1, NK cells, and B cells (Figure 9F). In contrast, MCP-counter analysis showed notable differences in B lineage and CD8^+^ T cells between the groups (Figure 9G).

**FIGURE 10:**
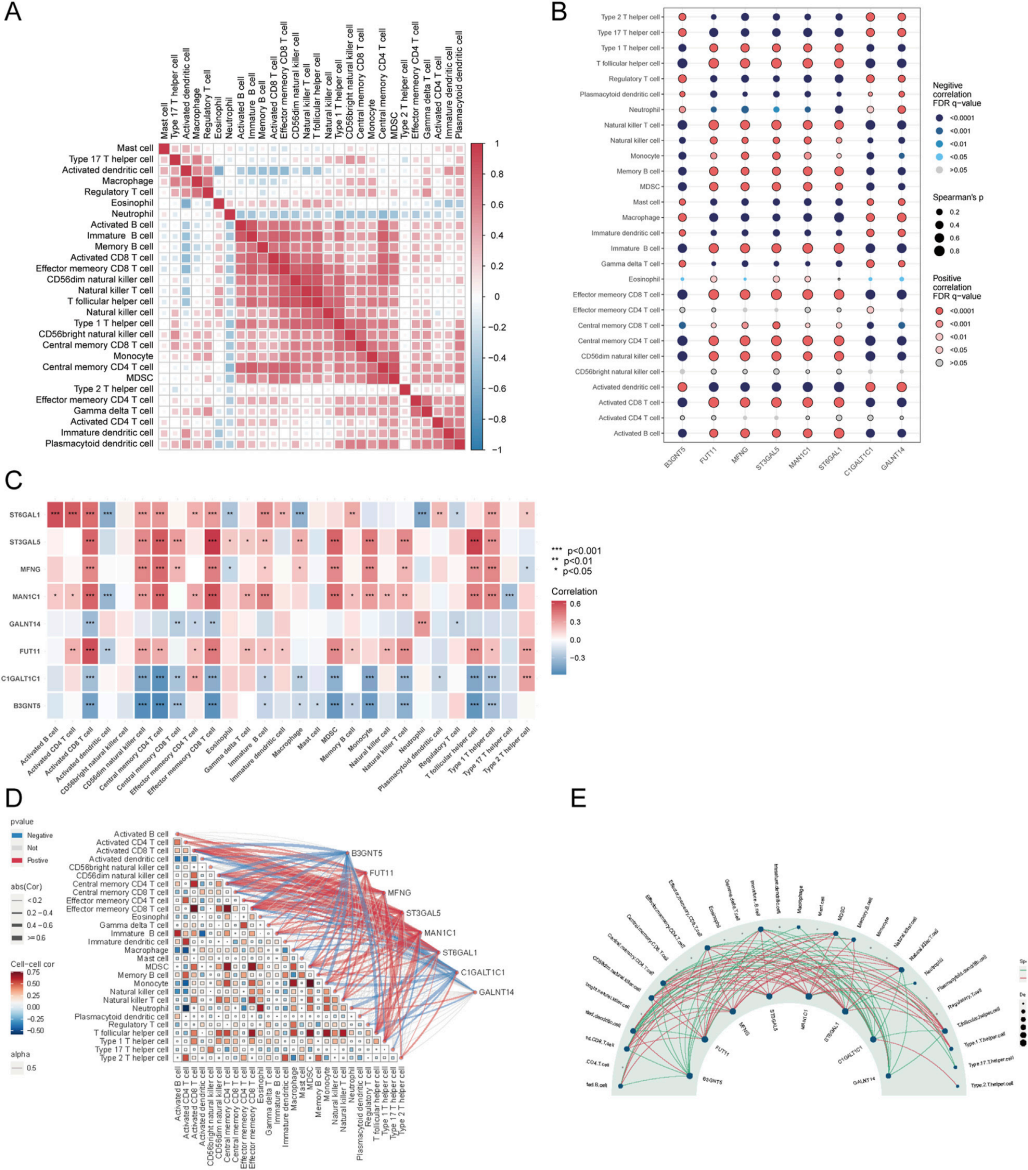
Correlation analysis of immune cells and GARGs. **(A)** Correlation matrix showing the relationships between proportions of immune cell types. Colour intensity represents the correlation strength, with red and blue indicating positive and negative correlations, respectively. **(B)** Dot plot showing the correlations between GARGs and immune cells. The dot size represents the strength of the correlation, and colour indicates the direction and significance level. **(C)** The heat map displays the correlations between GARGs and immune cell proportions, with red and blue indicating positive and negative correlations, respectively, and significance levels marked by asterisks. **(D)** A combined correlation plot illustrates the relationships between immune cells and their associations with GARGs. Red lines represent positive correlations, and blue lines represent negative correlations. **(E)** Circular plot highlighting the regulatory relationships between GARGs and immune cells. The size of each node represents the number of associated genes, with red lines indicating positive correlations and green lines indicating negative correlations.

Further examination of immune cell interactions in sepsis identified a strong synergistic relationship between central memory CD4^+^ T cells and immature B cells. Correlation analysis between immune cells and Golgi-related differential genes found that ST3GAL5 showed the strongest association with effector memory CD8^+^ T cells, whereas FUT11 was most strongly correlated with activated CD8 T cells (Figures 10A–E).

### 3.7 Identification of subtypes of the immune microenvironment in sepsis

**FIGURE 11:**
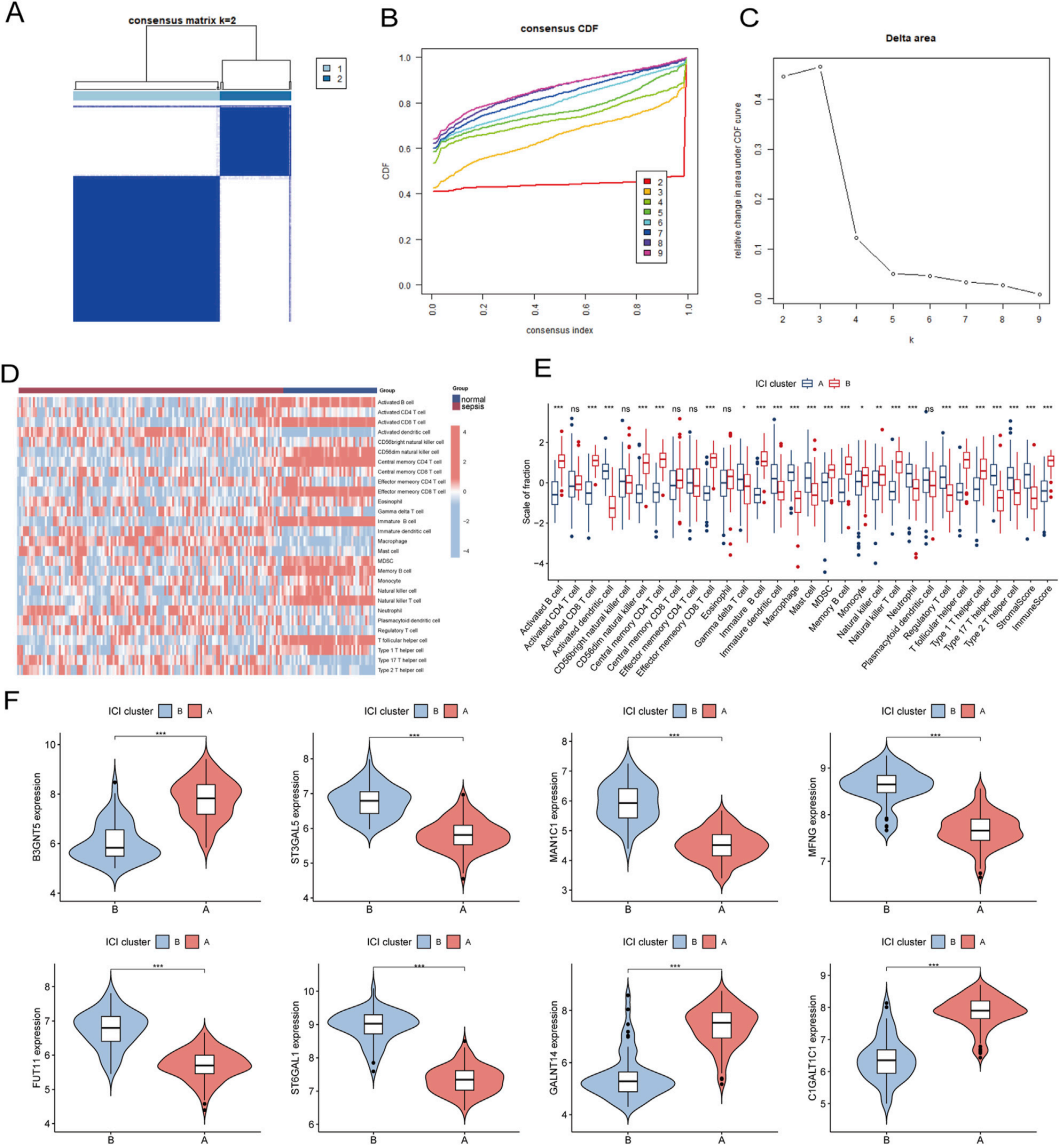
**(A)** Consensus clustering matrix when k = 2. **(B)** Consensus CDF curves when k = 2 to 9. **(C)** Relative alterations in CDF delta area curves. **(D)** The heat map shows ssGSEA scores for 28 immune cell subpopulations across the two subtypes. **(E)** Box plots comparing the infiltration levels of immune cell fractions between the two identified immune subtypes. **(F)** Split violin plots reveal the expression of 8 hub-GARGs between subtypes. Statistical significance: *P < 0.05, **P < 0.01, ***P < 0.001 and ns indicates no significance.

To investigate the expression patterns associated with the immune microenvironment in sepsis, we applied a consensus clustering algorithm. The consensus matrix was utilized as a similarity matrix to identify the final subtypes. Based on the consensus clustering results, including cumulative distribution function (CDF) plots, relative changes in the CDF curve area, and consensus clustering scores, we determined k = 2 as the optimal number of clusters. This grouping classified the 180 patients into two distinct subtypes, with 121 samples in subtype 1 and 59 samples in subtype 2 (Figures 11A–C). A comparative analysis of 28 immune cell subpopulations across subtypes revealed significantly higher infiltration levels of activated B cells, activated CD4 T cells, central memory CD4 T cells, effector memory CD4 T cells, regulatory T cells, and macrophages in subtype 1 (Cluster A) relative to subtype 2 (Cluster B) (Figures 11D, E). Furthermore, the expression levels of eight key genes were significantly different between the two immune subtypes (Figure 11F).

### 3.8 Construction of sepsis risk prediction diagnostic model and nomogram model

**FIGURE 12:**
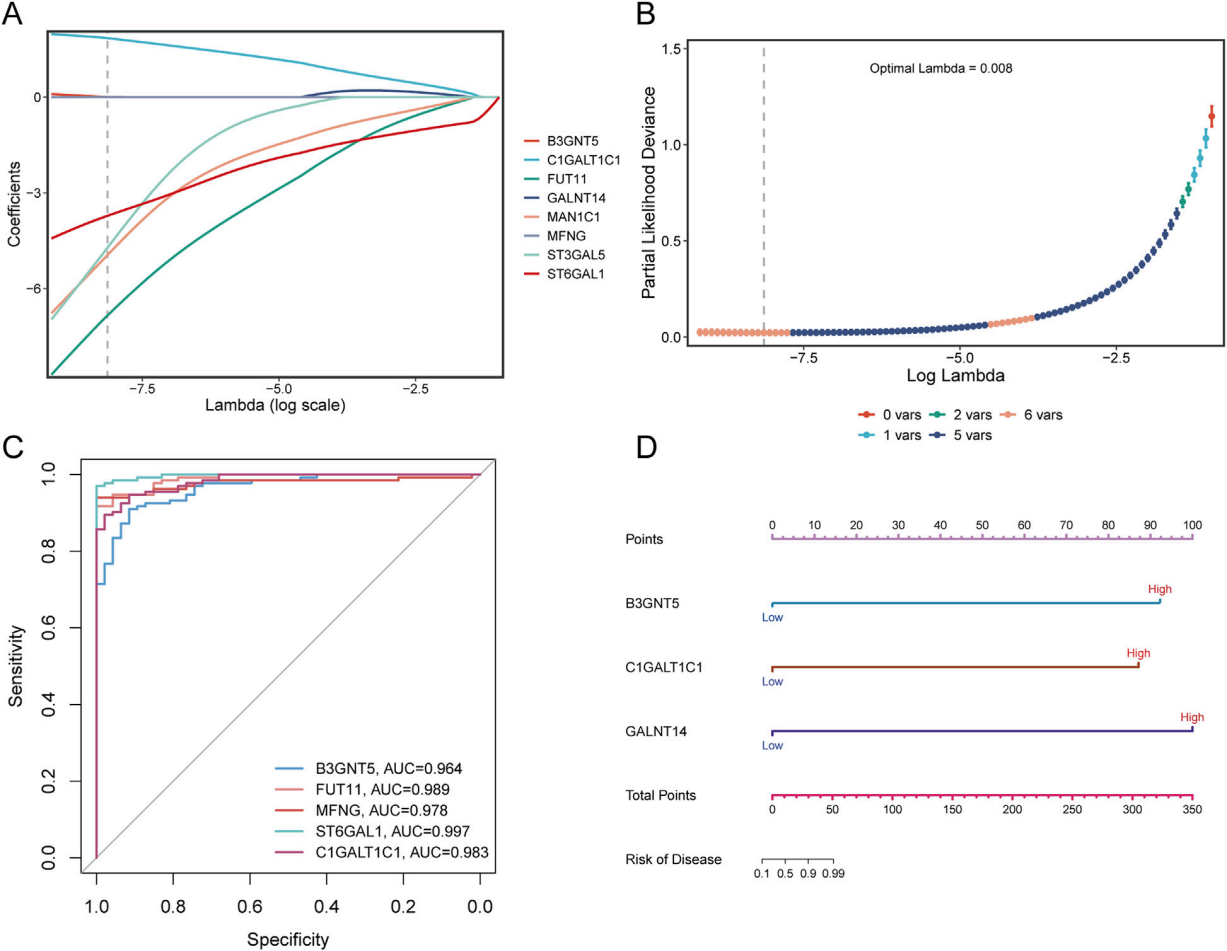
LASSO analysis and diagnostic evaluation of pivotal genes. **(A)** Path plot of LASSO coefficients for pivotal genes, illustrating the relationship between gene coefficients and the penalty parameter. **(B)** Cross-validation curve for LASSO regression, highlighting the optimal lambda value corresponding to the minimum partial likelihood deviance. **(C)** ROC curves demonstrate the diagnostic performance of pivotal genes, with AUC values indicating the predictive accuracy of each gene. **(D)** Nomogram model incorporating pivotal genes to estimate disease risk. The total points derived from individual gene scores are used to predict the likelihood of sepsis.

LASSO regression analysis was applied to eight Golgi-associated differential genes to identify those with prognostic potential (Figures 12A, B). This process resulted in the selection of five Golgi-associated differential genes: B3GNT5, FUT11, ST3GAL5, MAN1C1, ST6GAL1, and C1GALT1C1. ROC curves were generated for these five pivotal genes (B3GNT5, FUT11, MAN1C1, ST6GAL1, and C1GALT1C1) to evaluate their diagnostic performance (Figure 12C). The Area Under the Curve (AUC) values were as follows: B3GNT5 (0.964), FUT11 (0.989), MAN1C1 (0.978), ST6GAL1 (0.997), and C1GALT1C1 (0.983), indicating high diagnostic efficacy. Subsequently, a nomogram model was developed to predict the risk of sepsis (Figure 12D).

### 3.9 Validation of Golgi-related genes in sepsis

**FIGURE 13:**
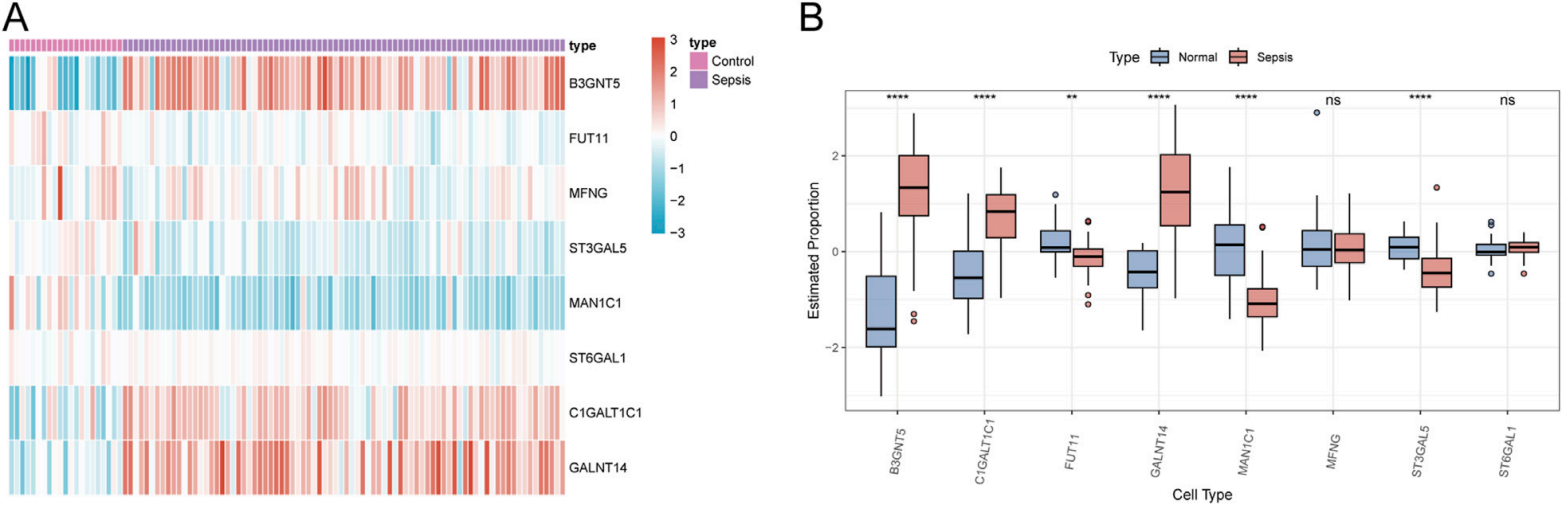
Validation of GARGs. **(A)** The heat map shows the expression patterns of GARGs in the validation dataset, comparing sepsis and control groups. Red indicates upregulated genes, and blue indicates downregulated genes. **(B)** Box plots compare the expression levels of GARGs between normal and sepsis samples in the validation dataset. Statistical significance is denoted as follows: *P < 0.05, **P < 0.01, ***P < 0.001, ****P < 0.0001, and ns indicates no significance.

To verify the robustness of our analysis on the sepsis dataset, we employed the dataset GSE26378, which comprises 21 normal and 81 sepsis patient samples, as a validation set. Within this set, we evaluated the expression of eight Golgi-associated genes using a logFC threshold of greater than 1 or less than −1. The expression trends of six Golgi-related genes (B3GNT5, FUT11, ST3GAL5, MAN1C1, C1GALT1C1, and GALNT14) aligned with the patterns observed in the hub genes from the initial sepsis dataset analysis (Figures 13A, B).

## 4 Discussion

Sepsis is a critical clinical condition characterized by a systemic inflammatory response initiated by an aberrant host reaction to infection, culminating in progressive multi-organ failure (Singer et al., 2016). The Golgi apparatus, a vital cytoplasmic organelle, plays a significant role in the post-translational modification, transportation, and sorting of lipids and proteins produced by the endoplasmic reticulum (ER). It ensures their delivery to specific cellular destinations through processes such as cytophagy and cytotoxicity (Casey et al., 2018). The dysfunction of the Golgi apparatus is increasingly acknowledged as a crucial contributor to the pathogenesis of various human diseases (Joshi et al., 2015). During sepsis, the interaction between pathogenic bacteria and cells induces structural and functional alterations in the Golgi apparatus, leading to a significant disturbance in the intracellular milieu. Studies have shown that during sepsis, interactions between pathogenic bacteria and host cells induce structural and functional alterations in the Golgi apparatus, disrupting intracellular homeostasis (Dusabimana et al., 2023). Golgi fragmentation facilitates the enhanced secretion of inflammatory mediators, thereby exacerbating the systemic inflammatory response (Choi et al., 2022). Beyond traditional inflammatory pathways, Golgi dysfunction has been linked to ER stress and oxidative damage, which are hallmarks of sepsis progression (Liu et al., 2021). In addition, Golgi interactions with other organelles are critical in maintaining cellular homeostasis, and their dysfunction may exacerbate the pathological process of sepsis. In sepsis, Golgi stress may lead to loss of mitochondrial membrane potential and accumulation of ROS, exacerbating cellular damage. Meanwhile, the Golgi and endoplasmic reticulum synergize in protein folding and secretion, and endoplasmic reticulum stress may further exacerbate its dysfunction by enhancing Golgi protein loading (Nagashima et al., 2020). These findings further reinforce our hypothesis that Golgi apparatus dysfunction is a critical factor in the pathogenesis of sepsis. By identifying key Golgi-associated genes, our study builds upon recent research, offering valuable insights and potential biomarkers for early diagnosis and targeted therapeutic interventions.

This study utilizes machine learning algorithms to develop decision-making models aimed at improving the diagnosis and treatment of sepsis. Acknowledging the pivotal role of the Golgi apparatus in sepsis pathogenesis, we conducted a bioinformatics analysis of Golgi-associated genes. Through a comprehensive examination of publicly available sepsis transcriptional datasets, we identified 661 upregulated and 561 downregulated differential genes. By intersecting the modular species from WGCNA with these differential and Golgi-related genes, 53 GARGs were identified. Among these, several genes showed potential as diagnostic markers or therapeutic targets. Studies have demonstrated that lipopolysaccharide (LPS) exposure escalates Golgi stress, fragmentation, and the production of related pro-inflammatory mediators both *in vivo* and *in vitro* (Dusabimana et al., 2023). Additionally, under conditions like oxidative stress and nutritional deficiencies, the Golgi apparatus can initiate a stress response, leading to the cleavage of Golgi structure-associated proteins (Li et al., 2019). The progressive damage to the Golgi apparatus in the rapid advancement of sepsis contributes to cell death and exacerbates the inflammatory response. Therefore, understanding the link between the Golgi apparatus and sepsis holds significant theoretical and clinical relevance, offering insights for the improved prevention and management of sepsis.

Sepsis is characterized by a dysfunctional immune response to pathogens, often leading to severe immune-mediated damage to vital organs and mortality. Our research identified significant disparities in the type and abundance of immune cell infiltration between sepsis patients and healthy controls, highlighting the critical role of immune cells in sepsis. In sepsis, the Golgi apparatus is central to orchestrating inflammatory responses and pathological processes through its regulation of immune cell function. Beyond serving as a central organelle for vesicular trafficking and protein and lipid transport, the Golgi also acts as a critical platform for innate immune signalling and the activation of downstream effectors (Tu et al., 2022). As a central organelle for protein processing and secretion, the Golgi apparatus orchestrates the release of inflammatory mediators and modulates the modification and expression of key receptors in immune cells. Furthermore, in close collaboration with the endoplasmic reticulum, the Golgi regulates intracellular stress responses and the activation of inflammatory vesicles, directly contributing to the functional execution of immune cell activities (Tao et al., 2020). Dendritic cells (DCs), as key players in innate immunity, are crucial for pathogen recognition, initiating immune responses, and regulating inflammation. Research indicates that preventing sepsis-induced DC apoptosis or enhancing DC functionality may enhance the long-term survival rates of sepsis patients (Efron et al., 2004). The Golgi apparatus plays a critical role in the assembly and trafficking of major histocompatibility complex (MHC) molecules and their associated antigenic peptides in DCs, thereby governing the efficiency of antigen presentation from DCs to T cells (Blander, 2023). Additionally, lymphocyte apoptosis has been recognized as a pivotal process in sepsis pathogenesis, contributing to immunosuppression associated with the disease (Lang and Matute-Bello, 2009). This phenomenon has been observed in both human patients and animal sepsis models (Hotchkiss et al., 2005). The development and function of lymphocytes are critically reliant on secreted proteins transported through ER to Golgi vesicular pathways (Yeerken et al., 2024). Experiments using the mouse cecum ligation and perforation (CLP) model have demonstrated diminished responses in naïve and memory CD4^+^ T and CD8^+^ T cells in sepsis survivors, increasing their vulnerability to new or recurrent infections (Serbanescu et al., 2016; Sjaastad et al., 2020). B-cells, with their diverse functional and phenotypic attributes, have been shown in mouse sepsis models to be essential for enhancing cytokine production, reducing bacterial loads, and improving survival via type I interferon signalling (Kelly-Scumpia et al., 2011). A reduction in immunoreactive B cells has been associated with a heightened risk of secondary infections post-sepsis (Delano and Ward, 2016). Golgi dysfunction contributes to dysregulated inflammatory responses and immunosuppression, potentially aggravating sepsis pathology. Thus, the Golgi represents an important avenue for understanding sepsis mechanisms and a potential target for therapeutic intervention.

In our study, we identified potential diagnostic markers for sepsis among Golgi-related genes and validated these findings using the GSE23678 dataset. The key genes distinguishing sepsis from healthy conditions include B3GNT5, FUT11, ST3GAL5, MAN1C1, C1GALT1C1, and GALNT14. B3GNT5 is pivotal in the biosynthesis of lactosylceramide sphingolipids and regulates embryonic antigens with lacto-series carbohydrate structures, suggesting its role as a biomarker in various tumours (Kim and Koo, 2019). Its overexpression, facilitating glycosylation-mediated protein stabilization, has been implicated in promoting tumorigenesis in breast cancer (Miao et al., 2022). The Fucosyltransferase (FUT) family, with FUT11 as a member, is involved in transferring fucose to glycoconjugates (Hwang et al., 2020), playing a significant role in tumorigenesis and progression. FUT11, specifically, is a Golgi-resident enzyme catalyzing the transfer of fucose to glycan entities, with implications for cancer and inflammation (Moriwaki and Miyoshi, 2010). FUT11, identified as a HIF-1-regulated gene in *Staphylococcus aureus* infections, may play a role in the host’s immune response (Beerlage et al., 2013). ST3GAL5, involved in sphingolipid GM3 synthesis, affects cell proliferation, differentiation, and integrin-mediated adhesion (Liu et al., 2022). MAN1C1, associated with cell viability and apoptosis, affects N-glycosylation and endoplasmic reticulum stress by regulating glycoprotein precursor processing (Li et al., 2018). MAN1C1 is involved in cellular immunity during chronic infections, and upregulation of its expression manipulates pathogens to evade immune recognition, inhibiting effective immune responses and clearance of pathogens (Hu et al., 2016). C1GALT1C1, a molecular chaperone in the endoplasmic reticulum, has been linked to tumorigenesis in several cancers (Shen et al., 2020). GALNT14 initiates O-glycosylation, influencing cancer cell proliferation, migration, and metastasis, with its expression levels and SNP genotypes predictive of cancer outcomes (Lin and Yeh, 2020). B3GNT5, FUT11, ST3GAL5, MAN1C1, C1GALT1C1, and GALNT14 are glycosyltransferases, representing a critical class ofenzymes involved in the glycosylation process (Lau et al., 2007). Glycosylation plays a pivotal role in sepsis by modulating the immune and inflammatory responses through bacteria-induced alterations in glycan structures. Conserved changes in the serum glycome during sepsis have been implicated as key mechanisms driving immune responses and pathological processes induced by bacterial infection, and these changes are closely associated with specific glycosylated proteins (Heindel et al., 2022). These findings highlight the potential of these genes as biomarkers for sepsis diagnosis and therapeutic intervention.

This study has several limitations. First, the data were derived from a publicly available database with a relatively small and insufficiently diverse sample size, potentially introducing selection bias and limiting the model’s generalizability to broader populations. While external datasets supported the reliability of the findings, the absence of validation using real clinical samples may impact the diagnostic markers’ performance in practical applications. Additionally, the identified key genes and immune infiltration patterns have yet to be further validated through molecular experiments, and their functional roles in sepsis pathogenesis require more in-depth investigation. Lastly, the model’s technical complexity presents challenges for clinical implementation, and its feasibility in real-world settings remains unverified. Future research should incorporate larger and more diverse clinical samples, conduct experimental validation, and address these limitations to enhance the credibility and translational potential of the results.

## 5 Conclusion

In summary, our research represents the inaugural systematic exploration of GARGsfor the diagnosis of sepsis. Utilizing bioinformatics analysis methods, we constructed a WGCNA-based co-expression network to identify Golgi-related hub genes within the context of sepsis. These findings potentially contribute to advancements in sepsis diagnostics and offer insights into the molecular mechanisms of genes associated with sepsis risk. Moreover, our study proposes new directions and targets for therapeutic intervention in sepsis, aiming to enhance treatment strategies and patient outcomes.

## Data Availability

The original contributions presented in the study are included in the article/supplementary material, further inquiries can be directed to the corresponding author.
